# A universal stress protein upregulated by hypoxia has a role in *Burkholderia cenocepacia* intramacrophage survival: Implications for chronic infection in cystic fibrosis

**DOI:** 10.1002/mbo3.1311

**Published:** 2022-12-09

**Authors:** Andrew O'Connor, Irene Jurado‐Martín, Margaritha M. Mysior, Anotidaishe L. Manzira, Joanna Drabinska, Jeremy C. Simpson, Mary Lucey, Kirsten Schaffer, Rita Berisio, Siobhán McClean

**Affiliations:** ^1^ School of Biomolecular and Biomedical Sciences University College Dublin Belfield Dublin Ireland; ^2^ UCD Conway Institute of Biomolecular and Biomedical Science Befield Dublin Ireland; ^3^ Cell Screening Laboratory, School of Biology and Environmental Science University College Dublin Belfield Dublin Ireland; ^4^ Department of Microbiology St. Vincent's University Hospital Elm Park Dublin Ireland; ^5^ Institute of Biostructures and Bioimaging National Research Council Naples Italy

**Keywords:** adaptation, *Burkholderia cenocepacia*, chronic infection, cystic fibrosis, intra‐macrophage survival, respiratory infection, universal stress proteins

## Abstract

Universal stress proteins (USPs) are ubiquitously expressed in bacteria, archaea, and eukaryotes and play a lead role in adaptation to environmental conditions. They enable adaptation of bacterial pathogens to the conditions encountered in the human niche, including hypoxia, oxidative stress, osmotic stress, nutrient deficiency, or acid stress, thereby facilitating colonization. We previously reported that all six USP proteins encoded within a low‐oxygen activated (*lxa*) locus in *Burkholderia cenocepacia* showed increased abundance during chronic colonization of the cystic fibrosis (CF) lung. However, the role of USPs in chronic cystic fibrosis infection is not well understood. Structural modeling identified surface arginines on one *lxa*‐encoded USP, USP76, which suggested it mediated interactions with heparan sulfate. Using mutants derived from the *B. cenocepacia* strain, K56‐2, we show that USP76 is involved in host cell attachment. Pretreatment of lung epithelial cells with heparanase reduced the binding of the wild‐type and complement strains but not the Δ*usp76* mutant strain, indicating that USP76 is directly or indirectly involved in receptor recognition on the surface of epithelial cells. We also show that USP76 is required for growth and survival in many conditions associated with the CF lung, including acidic conditions and oxidative stress. Moreover, USP76 also has a role in survival in macrophages isolated from people with CF. Overall, while further elucidation of the exact mechanism(s) is required, we can conclude that USP76, which is upregulated during chronic infection, is involved in bacterial survival within CF macrophages, a hallmark of *Burkholderia* infection.

## Graphical abstract

A *Burkholderia cenocepacia* universal stress protein encoded on the BCAM0276 gene (USP76) which is upregulated in chronic infection in people with cystic fibrosis (CF) and by hypoxia, is involved in attachment to human epithelial cells. It is required for growth in conditions associated with the CF lung, including acidic conditions and oxidative stress. Survival of usp76 deletion mutant was impaired in monocyte‐derived macrophages from CF patients, suggesting it is involved in bacterial survival within macrophages, a hallmark of *Burkholderia* infection.

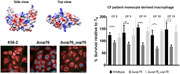

## 1 INTRODUCTION

Many opportunistic bacterial pathogens must adapt as they transition from their natural environment to the host to survive within the host and establish an infection. Changes in temperature, pH, osmolarity, and oxygen availability are among the stresses that bacteria must overcome when they colonize the mammalian niche. Universal stress proteins (USPs) are extensively expressed throughout nature from bacteria to archaea and eukaryotes in response to a wide variety of environmental conditions although they have not yet been identified in humans (O'Connor & McClean, 2017). This breadth of evolutionary range illustrates the importance of USPs in the resilience of organisms to survive in stressful environments, and while their role in bacterial cells is predominantly involved in the adaptive response to the changing environment, their contributions to bacterial pathogenesis cover a range of different mechanisms (O'Connor & McClean, 2017). In pathogens such as *Mycobacterium tuberculosis* and *Pseudomonas aeruginosa*, USPs are upregulated within macrophages and/or in response to low oxygen conditions, supporting survival under conditions of stress (Boes et al., 2006; Hingley‐Wilson et al., 2010; Schreiber et al., 2006), while an *Acinetobacter baumannii* USP has a protective role against low pH and contributes to pathogenesis (Elhosseiny et al., 2015). Recently, a USP was identified as a key determinant for the robustness of the nosocomial pathogen *Enterococcus faecium* during starvation (de Maat et al., 2020).

USPs were first identified in *Escherichia coli* K‐12 following exposure of cells to a variety of stresses, including heat shock, carbon and nitrogen starvation, and ultraviolet radiation (Nyström & Neidhardt, 1996). Expression of UspA in *E. coli* was independent of the stringent stress response transcriptional activators RelA/SpoT, RpoH, KatF, OmpR, AppY, Lrp, PhoB, and H‐NS (Nyström & Neidhardt, 1992). *E. coli uspA* gene deletion mutants were defective in survival over prolonged periods of growth under stress conditions such as induced peroxide stress and osmotic shock (Nyström & Neidhardt, 1994). *M. tuberculosis* USP Rv2623 contributes negatively to virulence by regulating its growth in the transition to latency (Drumm et al., 2009). There are six *usp* genes in the *E. coli* genome, while there are 10 *usp* genes encoded in the *M. tuberculosis* genome (O'Connor & McClean, 2017).

*Burkholderia cepacia* complex (Bcc) is a group of Gram‐negative bacteria that naturally occurs in soil and causes chronic opportunistic life‐threatening infections in people with cystic fibrosis (CF) and immunocompromised patients. It can also colonize pharmaceutical plants and contaminate pharmaceutical products and disinfectants (Vanlaere et al., 2009). It is highly antimicrobial resistant and once a chronic infection has been established, eradication is rare. Its capacity to colonize diverse and harsh niches is exemplary, and consequently, elucidation of its mechanisms of adaptation is essential Sass et al. (2013) identified a 50 gene locus that was dramatically upregulated under low oxygen conditions and designated the low oxygen activated (*lxa*) locus (Sass et al., 2013). We subsequently showed that 19 proteins encoded on the *lxa* locus showed increased abundance in late infection isolates from chronically colonized CF patients relative to early isolates (Cullen et al., 2018). These late chronic infection isolates also showed increased attachment to CF lung epithelial cells relative to their respective early isolates (Cullen et al., 2017). Importantly, all six USPs encoded on the *lxa* locus showed increased protein abundance with the time of colonization (Cullen et al., 2018) and five of which were regulated by the DNA mimic protein, Bnr1 (Dennehy et al., 2022). Among these *lxa*‐encoded *usp* genes, BCAM0276 encodes a UspA family stress protein which consistently showed increased abundance in the later isolates from two chronically colonized patients and was associated with increased gene expression (Cullen et al., 2018). Previously, this USP (USP76) was reported to be upregulated almost 60‐fold in *B. cenocepacia* strain J2315 in response to low oxygen (Sass et al., 2013), and up to 40‐fold in a comparative transcriptomic study of *B. contaminans* isolates from CF patients (Nunvar et al., 2016). There are 11 USPs in total encoded on the *B. cenocepacia* genome (Winsor et al., 2008), 10 of which are on chromosome 2. The role of USP76 or other USPs in Bcc is unknown, but this increased expression during chronic infection and under low oxygen conditions suggests that this gene may support the chronic persistence of *B. cepacia* complex during infection of the hypoxic CF lung. While USPs have been shown to protect bacteria from a range of environmental pressures and stresses including extreme temperature changes, antibiotic challenges, nutrient deprivation, and oxidative stress, to date there are limited published data on the role of USPs in chronic infection in CF.

CF lung disease is characterized by recurrent bacterial infections, exacerbations, and excessive pulmonary inflammation (Elizur et al., 2008; Roesch et al., 2018). Limited oxygen is another hallmark of the CF lung due to rapid oxygen consumption by microorganisms; neutrophils; impaired ventilation; and mucous plugging (Montgomery et al., 2017). These selective pressures in CF airways drive adaptation in colonizing bacteria, enabling them to overcome the CF lung microenvironments during infection. Moreover, pathogens such as Bcc can survive phagocytosis and even replicate within macrophages, contributing to chronic infection (Martin & Mohr, 2000; Rosales‐Reyes et al., 2012). To examine the role of USPs in this process, we compared two USPs encoded on the *lxa*‐locus, USP76, and USP92, which have comparable predicted sizes (Table 1). We demonstrate that these USPs are distinct and that USP76 is involved in supporting survival in human macrophage cells.

**Table 1:** USPs encoded on the *lxa* locus in *B. cenocepacia* J2315

Gene name	Predicted MW (Da)	Predicted pI	Predicted cellular Localisation (PSortb)
BCAM0276 (usp76)	17016.4	8.23	Cytoplasmic
BCAM0290	16139.1	4.61	Unknown
BCAM0291	30520.5	6.74	Cytoplasmic
BCAM0292 (usp92)	17743.9	4.82	Unknown
BCAM0294	30586.3	6.75	Unknown
BCAM0319	33622.2	6.86	Unknown

*Note*: Data taken from Burkholderia Genome database (www.burkholderia.com; Winsor et al., 2008).

## 2 EXPERIMENTAL PROCEDURES

### 2.1 Homology modeling

The homology model structures of USP76 and USP92 were obtained after consensus‐based sequence alignment using the HHpred tool. The best model template for USP76 was identified as the structure of the TeaD stress protein from the TRAP transporter TeaABC of *Halomonas elongata* (PDB code 3hgm, seqid 34.3%). For USP92, the best template was the BupsA stress protein from *Burkholderia pseudomallei* (PDB code 4wny, seqid 69.2%). Using these alignments, the homology models were built using the program MODELLER (Bitencourt‐Ferreira & de Azevedo, 2019). Electrostatic potential surfaces were computed using the program Chimera (Yang et al., 2012).

### 2.2 Bacterial strains and growth conditions

The strains and plasmids used in this study are listed in the Appendix (Table A1). Bacteria were routinely grown at 37°C in lysogeny broth (LB) with orbital shaking (200 rpm) unless otherwise stated. Antibiotics, when required, were added to reach final concentrations as follows: 50 μg/ml trimethoprim for *E. coli* and 100 μg/ml for *B. cenocepacia*, and 40 μg/ml kanamycin for *E. coli*.

### 2.3 Mammalian cell culture

CF epithelial cells, CFBE41o^−^ which are homozygous for the ΔF508 mutation of the CFTR gene were routinely cultured in collagen/fibronectin‐coated flasks as previously described (Cullen et al., 2017). The U937 macrophage cells were maintained as a suspension culture in complete RPMI medium (Sigma‐Aldrich) supplemented with 1 mM sodium pyruvate, 10 mM 4‐(2‐hydroxyethyl)‐1‐piperazineethanesulfonic acid (HEPES) buffer (pH 7.0–7.6), 1 mg/100 units streptomycin/penicillin, 10% (v/v) fetal bovine serum (FBS) and 5 g/L d‐glucose. U937 cells were plated in 24‐well plates and after 24 h were induced to differentiate by the addition of 15 ng/ml of phorbol 12‐myristate 13‐acetate (PMA) for 24 h in full RPMI medium.

### 2.4 Construction of ∆*usp* mutants and complementation

Targeted gene deletions of BCAM0276 or BCAM0292 in the *B. cenocepacia* strain K56‐2 were performed as described (Flannagan et al., 2008) and named as ∆*usp_76* and ∆*usp_92*, respectively. The amplicons used to construct the mutagenic plasmid were cloned into pGPI‐SceI‐2 digested with *EcoRI* and *NheI*, using triparental mating, followed by biparental mating to introduce the I‐SceI endonuclease. The screening was performed on the resulting colonies from bi‐parental mating to determine if successful gene deletion had taken place. Trimethoprim‐sensitive colonies were screened for gene deletion using the US forward DS reverse primers (Appendix 1, Table A2) and mutants were sent for sequencing (Eurofins Genomics Ltd) to confirm sequence deletion and stocks in glycerol prepared. To complement *B. cenocepacia* K56‐2Δ*BCAM0276*, wild‐type BCAM0276 was amplified from *B. cenocepacia* K56‐2 with the complementation primer pairs (Table A2), digested with the restriction enzymes *NdeI* and *XbaI* and ligated into similarly digested pMH447 (Hamad et al., 2012). The complementation plasmid was introduced into the mutant by conjugation. Once transferred into the target mutant strain the complementation vector integrates into the genome at aminoglycoside efflux genes (*BCAL1674‐BCAL1675*), due to sequence homology between the vector and target genome (Hamad et al., 2010). As before, pDAI‐Sce‐I was then introduced resulting in the replacement of BCAL1674‐1675 by BCAM0276. The complementation of the BCAM0276 gene was confirmed by polymerase chain reaction (PCR) and by phenotype analysis.

Following genetic manipulation of *B. cenocepacia* K56‐2 strain during gene deletion and subsequent complementation, the presence of the plasmid pC3 was confirmed using three sets of primers designed for genes *repA*, *oriC*, and *dopC*. The pC3 plasmid is a nonessential replicon of *B. cenocepacia* and encodes for a number of virulence factors (Agnoli et al., 2014), which can be lost during genetic manipulation. PCR was performed on confirmed mutants and complements using primers 3001/3002, 3003/3004, and 3005/3006.

### 2.5 Bacterial attachment to human CF epithelial cells

CFBE41o^−^ cells were seeded in wells of a 24‐well plate at a density of 4 × 10^5^ cells/well in antibiotic‐free medium and incubated overnight. The cells were then washed three times with phosphate‐buffered saline (PBS) and the mid‐logarithmic phase bacterial cultures (OD_600nm_ 0.6–0.8) were resuspended in MEM and added to each well at a concentration of 2 × 10^7^ CFU/well (MOI 50:1). The plates were centrifuged for 5 min and incubated for 30 min at 37°C, 5% CO_2_ to allow for bacterial adherence. Wells were then washed with PBS and lysis buffer (0.25% Triton X‐100 in PBS) added to each well for 20 min at room temperature (RT). Cell lysates were plated onto LB agar in duplicate and incubated at 37°C for 48 h. The resulting colonies were counted and the CFU/ml determined. For microscopic visualization of attachment, CFBE41o^−^ cells were seeded in chamber slides (LabTek™) and incubated with bacteria for 30 min, and washed with PBS as outlined above. CFBE41o^−^ cells and adherent bacterial cells were fixed using 3% w/v PFA (pH 7.2) for 10 min at RT, washed with PBS, and blocked with 5% bovine serum albumin (BSA) in PBS for 1 h at RT. Cells were then incubated with a rabbit anti‐Bcc antibody (courtesy of Prof U. Sajjan) in 1% BSA in PBS overnight at 4°C. Cells were then washed with PBS and incubated with an anti‐rabbit FITC conjugated antibody for 1 h at 4°C. Cells were then washed twice with PBS for 5 min. Nuclei were counterstained with 4′,6‐diamidine‐2′‐phenylindole dihydrochloride (DAPI) (VectaShield) and visualized using an Olympus FV‐1000 confocal microscope. Bacterial attachment was expressed as the number of bacteria per 100 cells in 10 randomly selected fields.

To assess the involvement of USP76 binding to heparan sulfate chains, attachment was determined in the presence and absence of heparanase to cleave host cell heparan sulfate. CFBE41o‐ cells were seeded in 24‐well imaging plates (Miltenyi Biotec) for 24 h. The medium was removed, and the wells were either incubated overnight in antibiotic‐free medium containing 200 ng/ml of preactivated human heparanase‐1 (Sigma‐Aldrich) (Reiland et al., 2004), or antibiotic‐free medium alone. The cells were then washed and incubated with bacteria at an MOI 50:1, the plates were centrifuged at 700 g for 5 min and incubated for 30 min at 37°C, 5% CO_2_. Wells were then washed with PBS and adherent bacterial cells were fixed and blocked as before. Cells were then incubated with a rabbit anti‐Bcc antibody (courtesy of Prof U. Sajjan) in 1% BSA PBS (1:1000 dilution) overnight at 4°C, followed by PBS washing and incubation with a DyLight® 488‐conjugated secondary goat anti‐rabbit antibody (Invitrogen) in 1% BSA PBS (1:1000 dilution) for 1 h, in the dark at RT. Epithelial cell plasma membranes and nuclei were counterstained with 0.5× CellMask™ Deep Red for 10 min, at 37°C in the dark, followed by a 15 min incubation with 0.25 µg/ml DAPI (Merck) at RT in the dark. Wells were finally covered with 1 ml PBS, and images were taken using an automated Opera Phenix™ High Content Screening (HCS) confocal microscope. Bacteria attached were enumerated by a researcher blinded to sample identity and attachment expressed as the number of attached bacteria per cell in five randomly selected fields in two biological replicates.

### 2.6 Cytokine secretion by CFBE41o^−^ cells following infection with *B. cenocepacia* strains

CFBE41o^−^ cells were seeded at a density of 4 × 10^5^ cells/well in a 24‐well plate in antibiotic medium for 24 h followed by a further 24 h in antibiotic and serum‐free medium before infection with *B. cenocepacia*. Overnight cultures of each strain were added to fresh LB medium and grown to an OD_600nm_ of between 0.4 and 0.8. Each strain (2 × 10^7^ CFU/ml, MOI 50:1) in MEM was added to each well in duplicate and incubated for 24 h at 37°C and 5% CO_2,_ then centrifuged at 315*g* for 12 min and supernatants were transferred to −80°C for storage before assay. Interleukin‐8 (IL‐8) and IL‐6 secretion was determined using OptEIA™ ELISA kits (Becton Dickenson) according to the manufacturer's instructions and the absorbance was measured at 450 and 570 nm on the Biotek Synergy H1 Multiplate reader.

### 2.7 Assessment of environmental stress responses

Several environmental stresses experienced during chronic infection within the CF lung and macrophage environment were tested on each of the *B. cenocepacia* strains.

#### 2.7.1 Growth under normal lab conditions

Overnight cultures (three biological replicates) were diluted to an OD_600_ of 0.1 in LB and 300 μl aliquots added in duplicate to the wells of a 96‐well round‐bottomed plate. The plates were incubated at 37°C, with orbital shaking and OD_600nm_ measured every 30 min for 24 h.

##### Controlled hypoxia at 6% O_2_

LB was equilibrated in a controlled hypoxia chamber (Coy Laboratories) at 6% oxygen for 24 h. Overnight cultures of the bacterial strains (10 ml) were centrifuged at 4000*g* for 15 min, resuspended in 10 ml fresh LB, transferred to 40 ml hypoxia equilibrated LB in 100 ml conical flask, and incubated statically at 37°C and 6% O_2_. Aliquots were sampled at 24‐h intervals over 8 days, serially diluted to 10^−7^ in Ringer's solution, plated onto LB agar plates in duplicate, and then incubated at 37°C for 48 h in normoxic conditions before enumeration.

##### Low pH

Overnight cultures were diluted 1:100 LB at either pH 4.5 or standard pH ~7.5 and 300 μl aliquots added in duplicate to the wells of a 96‐well round‐bottomed plate. The plates were incubated at 37°C, with orbital shaking and OD_600nm_ measured every 15 min for 20 h. Cell viability in low pH was also determined by inoculating a 100 ml flask of LB at pH 4.5 with 10 ml of an overnight culture and incubating at 37°C, 170 rpm, and plating hourly samples as described previously.

##### Oxidative stress

The effects of oxidative stress on the *B. cenocepacia* strains were assessed firstly by exposing the strains to a series of concentrations of hydrogen peroxide. Overnight cultures of each strain were diluted at 1:100 in fresh LB and 270 μl added in duplicate to corresponding wells in a 96‐well plate. A series of concentrations of H_2_O_2_ (0–1mM) were added to the wells and growth was determined in a Synergy H1 microplate reader at 37°C at medium shaking and OD_600nm_ measured every 15 min for 20 h. The viability of bacterial cells in an oxidative stress environment was also examined by treating cultures with 700 μM H_2_O_2_ and incubating at 37°C, 200 rpm. Hourly samples were serially diluted in Ringer's solution, and plated in duplicate to determine CFU/ml.

##### High osmolarity

The effect of high osmolarity was determined in each *B. cenocepacia* strain by adding 300 μl of a range of concentrations of NaCl (0%–5% w/v) or sucrose (0%–50%) to rows of a 96‐well plate. Overnight cultures were added to each well (3 μl) in duplicate and the plates were incubated in a Biotek Synergy H1 multiplate reader at 37°C for 24 h with OD_600nm_ measured every 15 min.

##### Heat stress at 42°C

The effect of heat on each *B. cenocepacia* strain was determined by transferring 10 ml of overnight cultures of each strain into 100 ml of prewarmed (42°C) LB and incubated at 42°C, 200 rpm. Hourly samples were diluted in Ringer's solution, plated, and enumerated after 48 h.

### 2.8 Bacterial uptake and survival in U937 macrophage cells

The internalization of *B. cenocepacia* strains by U937 macrophages was determined as previously described (McKeon et al., 2010). Briefly, overnight cultures of each strain were transferred to fresh LB and incubated until mid‐logarithmic phase (OD_600nm_ of 0.6–0.8) and diluted to an MOI of 5:1 (2.5 × 10^6^ CFU/ml) in RPMI medium (McKeon et al., 2010). U937 cells were seeded at 5 × 10^5^ cells/ml in a 24‐well plate, differentiated with PMA, washed twice with PBS, and 1 ml of each bacterial culture applied in duplicate. The CFU applied was confirmed by serial dilution of aliquots in Ringer's and plating in duplicate. The 24‐well plates were centrifuged at 1100*g* for 5 min, and incubated at 37°C, 5% CO_2_ for 2 h, before washing with PBS. Extracellular bacteria were killed with amikacin/ceftazidime (1 mg/ml each) for 2 h at 37°C, 5% CO_2_. Wells were then washed five times with sterile PBS before cells were lysed with 0.25% Triton X‐100 for 15 min and scraped, diluted, and plated as outlined above. The final wash was also plated to verify that all extracellular bacteria were killed. The % uptake was calculated as the intracellular CFU/ml at a 2‐h point relative to the CFU/ml applied to the cells at time zero. To examine intracellular survival of *B. cenocepacia* in U937 macrophages, bacteria were incubated with U937 as described above and after 2 h of incubation with each strain, the wells were washed PBS before the addition of RPMI amikacin/ceftazidime (1 mg/ml each) to each well and incubation for a further 2 h at 37°C, 5% CO_2._ The wells were subsequently washed twice with PBS and fresh antibiotics replaced and incubated at 37°C, 5% CO_2_ for up to 24 h. Cells were then washed, lysed, diluted, and plated as described above and CFU determined after 48 h.

### 2.9 Isolation of human peripheral mononuclear cells (PBMC) and differentiation

Age and gender‐matched adults with CF with no history of Bcc infection were recruited from St Vincent's University Hospital and blood samples were collected in ethylenediaminetetraacetic acid (EDTA) tubes, diluted with the same volume of Dulbecco's PBS (DPBS), and mixed by inversion. PBMCs were isolated by layering the blood over using Ficoll‐Paque plus (GE Healthcare) and centrifuging at 400*g* for 30 min at room temperature without breaking. Upper layers containing plasma and platelets were removed and the layers containing mononuclear cells were transferred to a fresh tube containing three volumes of DPBS. Cells were centrifuged at 400*g* for 15 min at room temperature, resuspended in 8 ml DPBS, and centrifuged at 100*g* for 10 min to remove platelets. The cells were resuspended in RPMI, cryopreserved at 2 × 10^6^ cells per ml in 40% FBS, 10% dimethyl sulfoxide, 50% RPMI (v/v), and stored in liquid nitrogen. Stored vials were revived as required and added to 5 ml RPMI‐1640 (Sigma‐Aldrich) medium (with no additives) and centrifuged at 100*g* for 15 min. The pellets were resuspended in 5 ml of RPMI‐1640, transferred to a T25, and incubated at 37°C, 5% CO_2_ for 2 h to allow the PBMCs to adhere to the flask. The cells were washed four times with warmed Mg^2+^ and Ca^2+^‐free Dulbecco's PBS. Full RPMI‐1640 medium containing 10% autologous Human serum (Sigma‐Aldrich), 10 mM HEPES (pH 7.0–7.6), 1 mg/100 units Penicillin/Streptomycin, 4.5 g/L d‐glucose, 1 mM sodium pyruvate and 1× non‐essential amino acids (Sigma‐Aldrich) were added to the flasks and the cells incubated with macrophage colony‐stimulating factor (25 ng/ml, MC‐SF) (MSC) at 37°C, 5% CO_2_ for 7 days with half media changes every 3 days. The cells were harvested from the T25 flask by addition of porcine trypsin/EDTA (Sigma‐Aldrich) in PBS and incubated at 37°C for 20 min. Cells were gently removed from the flask with a cell scraper and added to full RPMI medium and centrifuged at 252*g* for 10 min. Cells were resuspended in 1 ml of full RPMI medium, counted, and diluted to the required concentration of cells in a 24‐well plate. Plates were then incubated for 24 h at 37°C before use. Bacterial uptake and survival were then determined in the monocyte‐derived macrophage cells as described for U937 cells.

### 2.10 Statistical analysis

Statistical analysis of host cell attachment, cytokine secretion, and CF macrophage uptake was performed by one‐way analysis of variance (ANOVA) using Prism software with Bartlett's test. Two‐way ANOVA was used to analyze growth in the presence of organic and inorganic peroxide; growth under acidic conditions and CF macrophage survival data. Comparison of attachment in the presence and absence of heparanase was performed using two‐way ANOVA with Sidák's multiple comparison test in Prism to compare individual strains. Statistical analysis of virulence, survival at 8 days in hypoxic conditions, endpoint OD_600nm_ in acidic conditions, and U937 internalization was performed using Student's *t* test.

Assessment of antibiotic resistance, mucoidy, motility, and biofilm formation was performed as described in Appendix 3.

## 3 RESULTS

### 3.1 Structural features of USP76 and USP92

The BCAM0276 and BCAM0292 genes are two of six USP‐encoding genes located on the *lxa* locus (Sass et al., 2013). We previously reported that USP76 and USP92, showed up to 6‐ and 16‐fold increased abundance, respectively, from early to late infection in two sets of sequential isolates and were selected for further investigation (Cullen et al., 2018). Moreover, we showed that the abundance of these was reduced by 1.66‐ and 4.5‐fold when BCAS0292, which encodes the DNA mimic protein Bnr1, was deleted (Dennehy et al., 2022).

**Figure 1:**
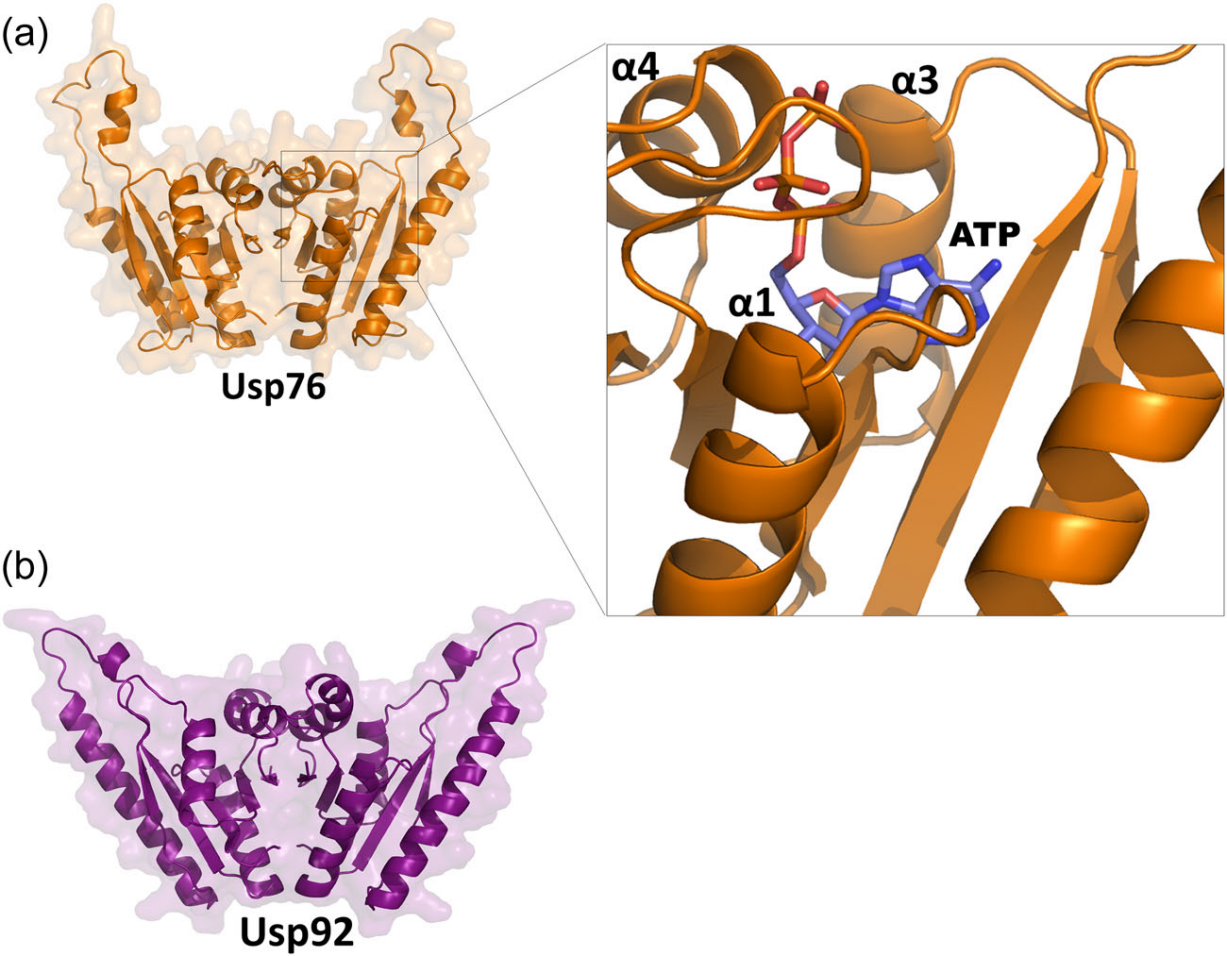
USP76 and USP92 share similar dimeric structural organization. Cartoon and surface representations of (a) USP76 and (b) USP92 homology models. The inset of panel A shows a detail of ATP binding mode. ATP, adenosine triphosphate.

Both USP76 and USP92 belong to the UspA protein family (PF00582) and contain a single UspA domain from residue 1 to 145 (Sousa & McKay, 2001). To identify structural factors of USP76 and USP92 to determine potential roles in pathogenesis and identify any structural differences, we used homology modeling and analyzed the resulting structures. Both structures are highly reliable, given the high sequence identities with their template structures (33.4% and 69.2%, respectively [see Methods]). Both protein structures share a similar dimeric organization (Figure 1), consistent with their 41.8% sequence identity and 58% homology (Altschul, 1997) which are above the expected threshold for protein similarity (Rost, 1999). Each monomer is composed of an open‐twisted, five‐strand parallel β‐sheet, sandwiched by two α‐helices on each side of the sheet (α1–α4 in Figure 1) and presents structural features of adenosine triphosphate (ATP)‐binding proteins. In both models, a wide ATP binding cleft runs adjacent to the central β‐sheet and contacts the α‐helices α1, α3, and α4 of each monomer (Figure 1).

**Figure 2:**
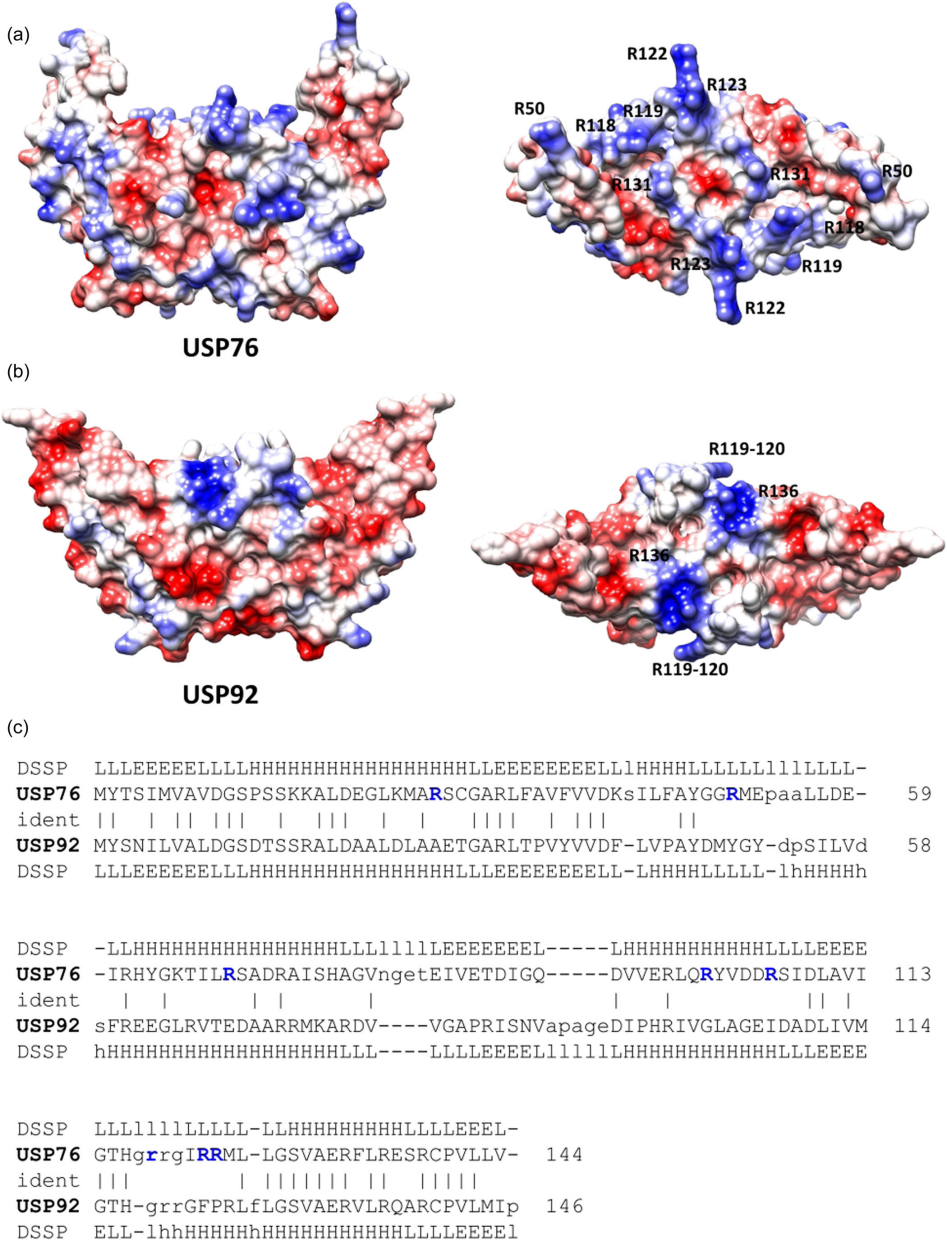
Comparison of electrostatic potential surfaces of USP76 and USP92. Electrostatic potential surfaces of (a) USP76 and (b) USP92. In each panel, side and top views are reported on the left and right sides, respectively. The main residues contributing to the electrostatic potential are labeled. (c) Structure‐based sequence alignment and secondary structure assignment (DSSP database) as computed by DALI. Non‐conserved arginine residues of USP76 are shown in blue.

Strong differences between the two proteins are evident when electrostatic potential surfaces are compared. Indeed, an overall negative electrostatic potential surface characterizes USP92, with only two positively charged patches due to Arg119‐120 and Arg136 (Figure 2a,b). Consistent with a higher pI value of USP76 (pI = 7.8) compared to USP92 (pI = 5.0), the electrostatic potential surface of USP76 presents several clusters of positively charged residues on the entire surface of the protein, due mostly to arginine residues (Figure 2).

### 3.2 The ∆*usp76* mutant has a reduced attachment to host cells

**Figure 3:**
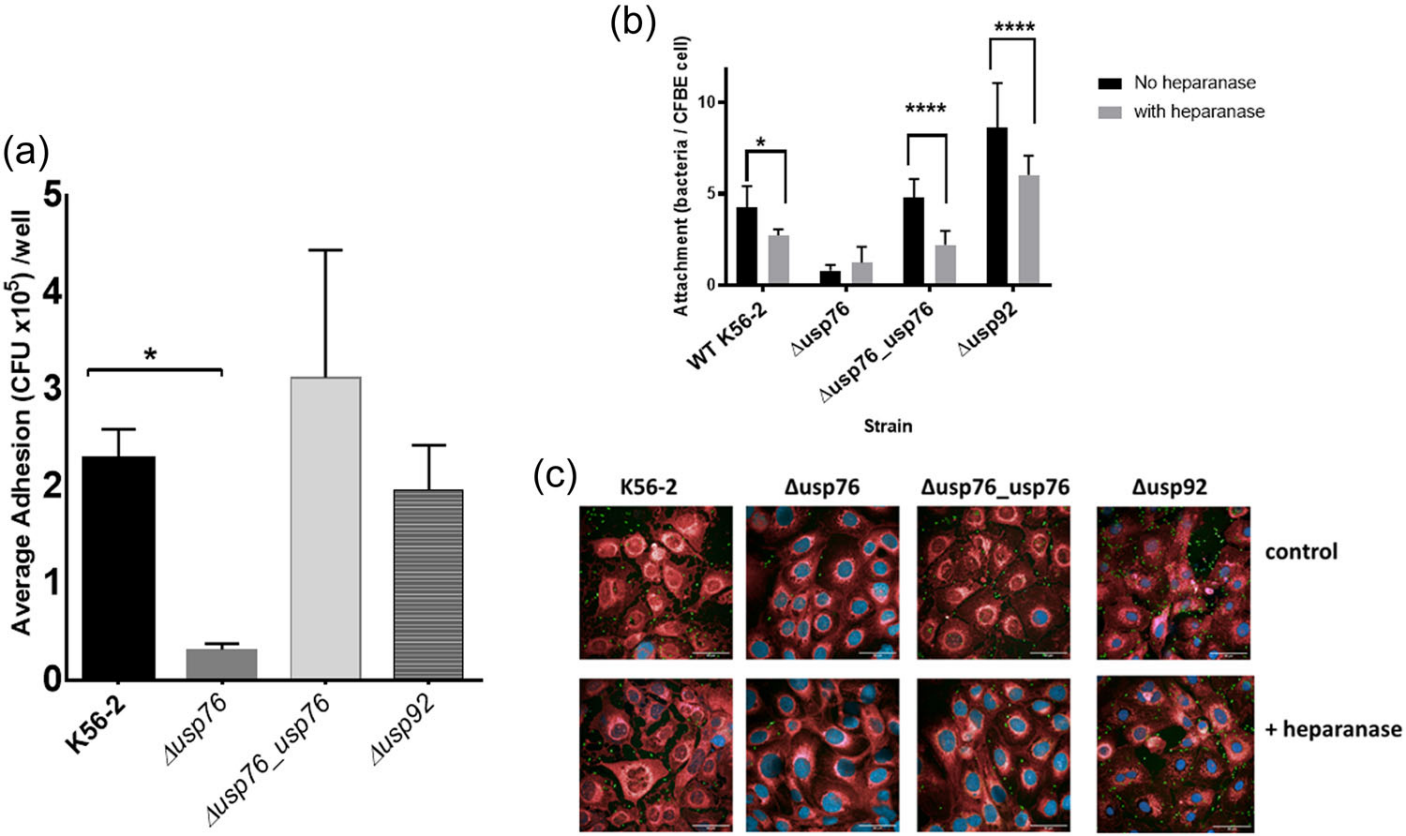
Comparison of the bacterial attachment of Δ*usp76* and Δ*usp92* mutants to CFBE41o^−^ cells. (a) Comparison of the attachment of WT K56‐2 strain, Δ*usp76* and Δ*usp92* mutant to CFBE41o^−^ over 30 min by microbiological plating at an MOI of 50:1. Data represent the mean CFU/ml for each strain in three independent experiments. Error bars represent the standard error. *Signifies a statistically significant difference in attachment of the ∆*usp76* mutant as determined by one‐way ANOVA, *p* = 0.0117. (b, c) Effect of heparan sulfate cleavage on attachment of WT, Δ*usp76*, Δ*usp76_usp76*, and Δ*usp92* strains as determined by confocal microscopy. (b) Data represent the mean number of bacteria per CFBE41o^−^ cell per strain in 30 min in five randomly selected fields of view in the absence (black) or presence (grey) of enzyme pretreatment in two independent biological replicates. Error bars represent the standard deviations of 10 replicate values in each condition. Statistically significant difference by two‐way ANOVA with a Sidák's multiple comparison test (*p = 0.0281; ****p < 0.0001). (c) Representative images of epithelial cells stained with CellMask™ Deep red and counterstained with DAPI with bacterial strains stained with anti‐Bcc polyclonal antibody and DyLight™ 488 conjugated anti‐rabbit IgG. ANOVA, analysis of variance; DAPI, 4′,6‐diamidino‐2‐phenylindole; IgG, immunoglobulin G.

We previously showed that the attachment of sequential *B. cenocepacia* isolates to CF epithelial cells, CFBE41o^−^, increased over the time of chronic infection (Cullen et al., 2017). Given the observed increase in BCAM0276 gene expression and a corresponding increase in USP76 protein abundance (Cullen et al., 2018), the effect of the deletion of BCAM0276 on attachment to CFBE41o^−^ cells was examined using the genetically amenable strain, *B. cenocepacia* K56‐2. The resulting mutant which we refer to throughout as the ∆*usp76* mutant showed 90% reduced attachment to CFBE41o^−^ cells (Figure 3a, *p* < 0.05), which was restored to wildtype levels in the Δ*usp76_usp76* complement strain (*p* = 0.1636). In contrast, the ∆*usp92* mutant (BCAM0292 gene deletion mutant) showed comparable attachment to CFBE41o^−^ cells.

This led us to speculate that USP76 protein may be involved in host–pathogen interactions with the surface arginine residues on USP76 playing a role in host extracellular matrix attachment, via electrostatic interactions with sulfonate and carboxylate groups of heparan sulfate proteoglycan on the surface of epithelial cells. To test this hypothesis, we treated CFBE41o^−^ cells with heparanase to remove heparan sulfate as a potential ligand and measured attachment by confocal microscopy. Heparanase pretreatment significantly reduced the binding of all strains that express USP76 (WT strain [*p* = 0.0281]; Δ*usp76_usp76* complement strain [*p* < 0.0001]; and Δ*usp92* strain [*p* < 0.0001]) but not the Δ*usp76* mutant strain (*p* = 0.8859) (Figure 3b,c), which supports the finding that the surface arginines on USP76 are directly or indirectly involved in interactions with heparan sulfate on the surfaces of epithelial cells.

### 3.3 The ∆*usp76* mutant elicits a reduced cytokine response

**Figure 4:**
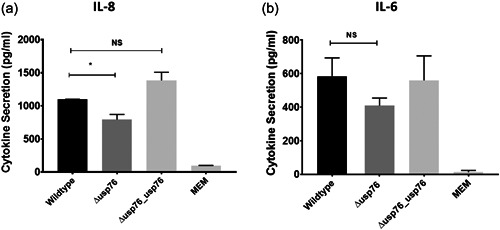
Effect of deletion of Δ*usp76* on cytokine response. IL‐8 (a) and IL‐6 (b) cytokine secretion from CFBE41o^−^ following infection with the WT, the Δ*usp76* mutant, the Δ*usp76*_*usp76* complement strains, and negative control (MEM only). Bars represent the mean level of detected cytokine in triplicate measured 24 h after exposure in two independent experiments. Error bars represent the standard deviation. Statistical analysis was performed using one‐way ANOVA, *p = 0.0135. ANOVA, analysis of variance; IL, interleukin.

Given that deletion of the BCAM0276 gene resulted in reduced attachment to CFBE41o^−^ cells, we focussed on USP76 to probe its role in pathogenesis further. We measured the secretion of IL‐8 and IL‐6 cytokines by CFBE41o^−^ cells 24 h after exposure to *B. cenocepacia* strains to investigate whether the absence of USP76 impacted host response. IL‐8 secretion was impaired in response to the ∆*usp76* mutant strain (Figure 4a; *p* = 0.0135), most likely due to reduced cellular attachment; while no statistically significant effect on IL‐6 relative to wild‐type was observed.

### 3.4 USP76 is required for survival in low oxygen and under an oxidative environment

**Figure 5:**
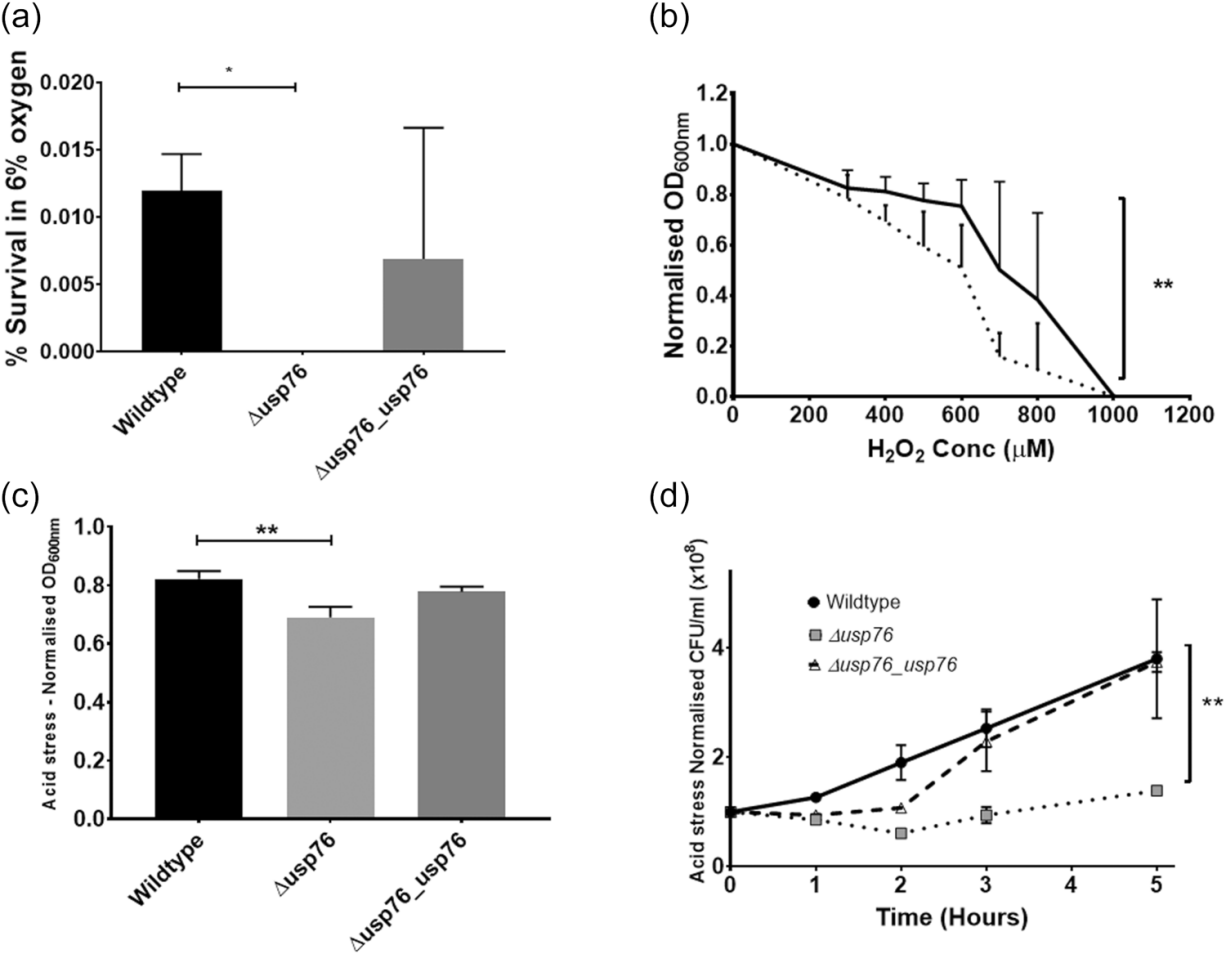
Effect of USP76 on survival or growth to environmental conditions. (a) Mean percentage survival of the WT, the ∆*usp76* mutant, and the Δ*usp76_usp76* complement strains after 8 days of incubation in 6% oxygen in a controlled hypoxia chamber determined in three independent experiments. Error bars represent the standard error; *p = 0.0258. (b) Mean OD_600nm_ values of the WT (solid line) and ∆*usp76* mutant (dashed line) strains following incubation with a range of concentrations of H_2_O_2_ for 20 h. Data displayed were normalized relative to treatment‐free control and represent duplicate values of three independent experiments, **p = 0.0016. (c) Mean normalized endpoint OD_600nm_ values for each strain WT, the ∆*usp76* mutant, and Δ*usp76_usp76* complement following 20 h incubation in pH 4.5 LB versus LB medium at pH 7. Data represent the mean normalized absorbance from three independent experiments, and error bars represent the standard error. Statistical analysis was performed by *t*‐test, **p = 0.0068. (d) Normalized survival (CFU/ml) over time for each strain, the ∆*usp76* mutant and the Δ*usp76_usp76* complement strains compared to *T*_0_ following incubation in LB pH 4.5. Data represent the mean normalized CFU/ml from three independent experiments, error bars represent the standard error. Statistical analysis by two‐way ANOVA, **p = 0.0019. ANOVA, analysis of variance; LB, lysogeny broth.

Deletion of the BCAM0276 gene did not have any impact on the growth of the ∆*usp76* mutant under normal lab conditions (LB, 37°C with shaking) when compared to the wild‐type strain. Previous studies on bacterial USPs highlight roles in growth or survival during challenging environmental conditions (O'Connor & McClean, 2017), and given the increase in BCAM0276 gene expression and abundance of USP76 in *B. cenocepacia* chronic infection isolates (Cullen et al., 2018), the response of ∆*usp76* under various environmental pressures experienced during chronic infection was examined. It has been shown that the *lxa* locus, including the BCAM0276 gene, was dramatically upregulated in response to low oxygen (Sass et al., 2013) therefore it was important to understand if USP76 was involved in survival in response to low oxygen. The K56‐2 strain was unable to grow in oxygen environments lower than 6% oxygen and survival was considerably impaired at 6% oxygen (Figure 5a). When exposed to 6% oxygen in a controlled hypoxia chamber, no ∆*usp76* mutant cells survived to Day 8, indicating that this gene is critical to extended survival under low oxygen. Complementation of the gene in the Δ*usp76_usp76* strain restored survival to near WT levels.

The CF lung is also characterized by constant inflammation and the production of reactive oxygen species due to persistent bacterial infections. The impact of the oxidative environment on the survival of the ∆*usp76* mutant was examined using hydrogen peroxide. Growth of the ∆*usp76* mutant was reduced when cultured in the presence of hydrogen peroxide (*p* = 0.0016, Figure 5b) for 20 h.

### 3.5 USP76 is required for growth and survival at acidic pH

Several bacterial USPs support survival under acidic conditions. The growth of the *∆usp76* mutant in LB at low pH (pH 4.5) was reduced (*p* < 0.003) over 20 h relative to WT when normalized for growth in LB at physiological pH (~pH 7.4, Figure 5c). Growth of the Δ*usp76_usp76* complemented strain was comparable to WT levels. When the strains were plated to determine the viable CFU remaining after exposure to acidic pH over a range of time points, it was apparent that the viability of the K56‐2 strain following growth in acidic conditions was also dependent on USP76 expression (Figure 5d, *p* = 0.0092). The Δ*usp76_usp76* complement strain showed an ability to recover, reaching WT levels within 5 h of exposure to acidic pH.

### 3.6 USP76 has no impact on antibiotic susceptibility or response to osmotic stress

**Figure A2:**
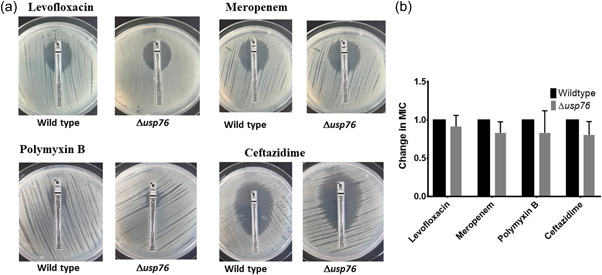
Antibiotic susceptibility testing via MICs as determined by ETEST® antibiotic susceptibility. (a) Representative images of ETEST results on WT and Δ*usp76* strains. (b) Calculated fold changes in MIC for WT and Δ*usp76* for the antibiotics Polymyxin B, Ceftazidime, Levofloxacin, and Meropenem using the Biomerieux ETEST®. Data shown represent the mean‐fold change in MIC from three independent occasions. Error bars represent the standard error. MIC, minimum inhibitory concentrations.

**Figure A3:**
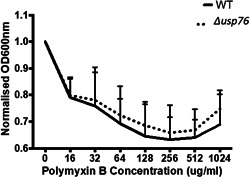
Normalized endpoint OD_600nm_ of WT and Δ*usp76* following 20 h incubation in various concentrations of Polymyxin B. Normalized endpoint OD_600nm_ of WT and Δ*usp76* incubated for 20 h in concentrations of Polymyxin B (0–1024 μg/ml). Data shown represent the mean OD_600nm_ from three independent occasions. Error bars represent the standard error. WT, wild‐type.

BCAM0276 did not have any role in survival in response to antibiotic challenges encountered during treatment for CF infection. Growth following exposure to antibiotics with a range of modes of action (Meropenem, Levofloxacin, Ceftazidime, and Polymyxin B) was comparable for the WT and the *∆usp76* mutant strains (*p* = 0.909) (Appendix 2, Figure A2, A3).

**Figure A4:**
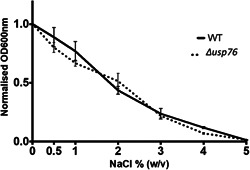
Endpoint normalized OD_600nm_ of WT K56‐2 and Δ*usp76* following a 24 h incubation with concentrations ranging from 0% to 5% W/V NaCl. Normalized endpoint absorbance values measured at 24 h incubation of WT K56‐2 and Δ*usp76* at 37°C in various concentrations of NaCl ranging from 0% to 5% w/v. Values displayed have been normalized to 0% W/V NaCl (LB only with culture) and represent the average of 3 independent occasions. Statistical analysis determined by ANOVA (*p* = 0.72). ANOVA, analysis of variance; LB, lysogeny broth; WT, wild‐type.

The CF lung and the airway surface liquid also represent high salt environments with 1% w/v NaCl concentrations reported for the ASL of people with CF compared to 0.7% in healthy controls (Grandjean Lapierre et al., 2017). This creates osmotic stress on any pathogen colonizing the CF lung. The growth of the ∆*usp76* mutant was comparable to that of the WT strain over a range of salt concentrations (0%–5% NaCl, *p* = 0.72) indicating that USP76 does not play a role in the salt tolerance of *B. cenocepacia* (Appendix 2, Figure A4).

### 3.7 Deletion of *usp76* does not affect mucoidy, motility, or biofilm formation

**Figure A5:**
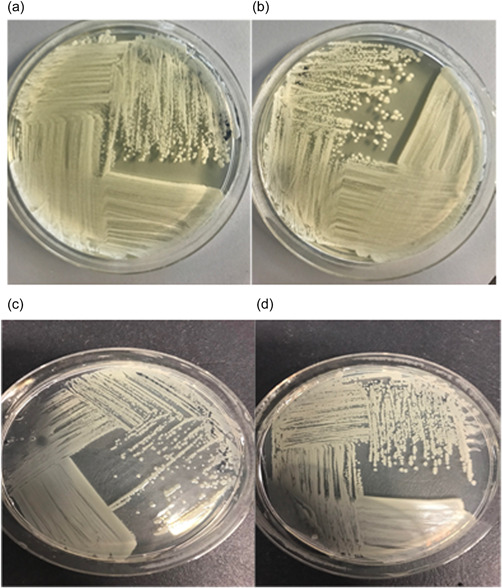
Representative images of the mucoid phenotype of WT and Δ*usp76* strains in normal oxygen conditions and at 6% O_2_. EPS production of WT (a) and Δ*usp* (b) incubated on YEM agar for 5 days at 37°C in either normal oxygen conditions (a, b) or 6% O_2_ (c, d). Images represent mucoid phenotype on 1 of 3 independent occasions. EPS, exopolysaccharides; YEM, Yeast extract mannitol.

**Figure A6:**
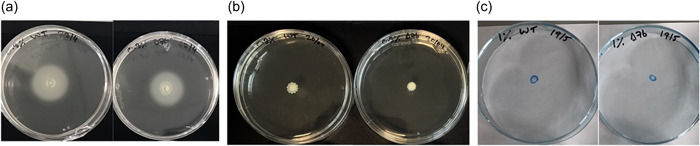
Motility of WT and Δ*usp76*. WT, wild‐type. (a) Swimming of strains on 0.3% w/v LB agar. Representative images of 3 independent occasions of the assessment of swimming of WT and Δusp76 on 0.3% w/v LB agar incubated for 16–18 hours. (b) Representative images of the assessment of swarming of WT and Δusp76 on 0.5% w/v LB agar. Representative images of 3 independent occasions of the assessment of swarming of WT and Δusp on 0.5% w/v LB agar incubated for 16–18 hours. (c) Representative images of the assessment of twitching of WT and Δusp76 on 1.0% w/v LB agar. Representative images of 3 independent occasions of the assessment of twitching of WT and Δusp stained with Coomassie blue following incubation on 1.0% w/v LB agar for 16–18 hours.

**Figure A7:**
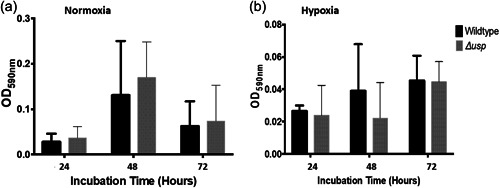
Determination of biofilm formation of WT and Δ*usp76* mutant over a period of 72 h in normoxic and hypoxic conditions. Average biofilm formation for the WT and the Δ*usp76* mutant strain as determined by measuring OD_590nm_ of resolubilized, crystal violet stained biofilms in normal oxygen (a) and hypoxic (6% O_2_) (b) conditions measured every 24 h for 72 h. Data represent the mean OD_590nm_ from three independent occasions. Error bars represent the standard error of each mean. WT, wild‐type.

Alterations in mucoid phenotype have been associated with chronically infecting Bcc isolates (Zlosnik et al., 2008), however, deletion of the *usp76* gene did not impact exopolysaccharides (EPS) production when grown on Yeast extract mannitol (YEM) agar plates in either normoxic or hypoxic conditions (Appendix 1, Table A3; Appendix 2, Figure A5). Motility was comparable between the WT and the *∆usp76* mutant strain (Appendix 2, Figure A6). Non‐statistically significant differences were observed for the motility phenotype when analyzed by ANOVA, swimming *p* = 0.9720, swarming *p* = 0.2044, and twitching *p* = 0.4226. In addition, the ability of *B*. *cenocepacia* to form biofilms was not affected by the presence of the *usp76* gene (Appendix 2, Figure A7) under normal oxygen conditions or hypoxic conditions.

### 3.8 USP76 is required for the survival of *B. cenocepacia* in CF macrophages

**Figure 6:**
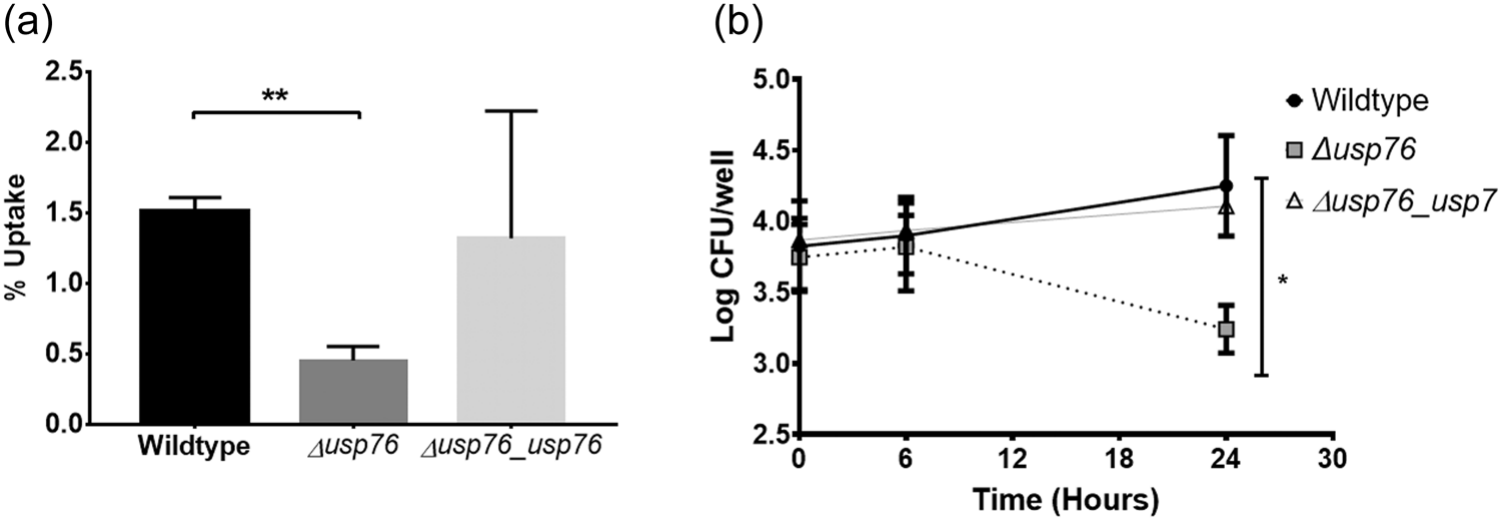
Uptake and survival of WT, the *∆usp76* mutant, and the Δ*usp76_usp76* complement strains in U937 macrophage cells. (a) Uptake of the WT, the *∆usp76* mutant, and the Δ*usp76_usp76* complement strains by differentiated U937 macrophage cells. Data represent the intracellular uptake of the individual strains as a % of bacterial cells applied in three independent experiments. Error bars represent the standard error of the mean. **Statistically significant difference relative to the WT as determined by Student *t* test, *p* = 0.002); (b) Survival of the ∆*usp76* mutant and the Δ*usp76_usp76* complement strains in U937 macrophage cells over time. Data represent the mean log_10_ of CFU/ml from three independent experiments; error bars represent the standard error of the mean. *Statistically significant difference relative to the WT determined by two‐way ANOVA (*p* = 0.0378). ANOVA, analysis of variance; WT, wild‐type.

**Figure 7:**
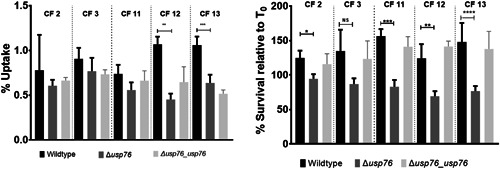
Uptake and survival of the WT, the ∆*usp76* mutant, and the Δ*usp76_usp76* strains in PBMC‐derived macrophage from people with CF. (a) % uptake and (b) 24 h survival of the WT (black), the ∆*usp76* mutant (grey bars) or the Δ*usp76_usp76* complement strain (grey patterned bars). The data represent the means of three independent experiments and error bars represent the standard error of each mean. Statistically significant difference relative to the WT as determined by one‐way ANOVA (**p = 0.0018; ***p = 0.0004). (b) Survival of the wild type, *∆usp76* mutant, and the Δ*usp76_usp76* complement strains in monocyte‐derived macrophage cells. Normalized data represent the mean log of CFU/ml from three independent experiments, relative to time zero. Error bars represent the standard error of the mean. Statistically significant difference relative to the WT determined by one‐way ANOVA as follows: CF3: *p* = 0.0403; CF 10, *p* = 0.0016; CF 11 *p* = 0.0025; CF12, *p* < 0.0001. ANOVA, analysis of variance; PBMC, peripheral blood mononuclear cell; WT, wild‐type.

Bcc can survive and replicate within macrophages (Martin & Mohr, 2000; Rosales‐Reyes et al., 2012; Vergunst et al., 2010) allowing the organism to evade both host immune response and antibiotic treatments. Given that the pH of the intramacrophage phagosome is estimated to be between 6.2 and 4.5, and that we have shown that the ∆*usp76* mutant is more susceptible to low pH and oxidative induced stress, we wanted to investigate whether USP76 might contribute to the survival of *B. cenocepacia* inside macrophages. First, we examined whether the intracellular uptake into the U937 macrophage‐like cell line was altered by the presence or absence of USP76 and found that there was a 60% reduction in the number of the *∆usp76* mutant cells that were internalized by the U937 line compared to WT (Figure 6a, *p* = 0.002). Phagocytosis of the Δ*usp76_usp76* complement strain was equivalent to that of WT. Survival of the ∆*usp76* mutant within U937 macrophage cells was also significantly impaired over 24 h (Figure 6b, *p* = 0.0378). In contrast, survival of the complemented strain was restored to WT levels. Acidification is impaired in CF macrophages due to the dysfunctional CFTR (Di et al., 2006), consequently, to examine whether this had relevance in the CF context, we sought ethical approval to investigate the survival of the strains in PBMC‐derived macrophages from 12 people with CF. PBMC samples from five subjects (all male) could be successfully differentiated into macrophage cells in sufficient numbers to be suitable for use in uptake and intracellular assays. The uptake of the ∆*usp76* mutant strain by PBMC‐derived macrophage from two patients (CF 11 and CF 12) was significantly reduced (Figure 7a, **p = 0.0018, ***p = 0.0004) when compared to the WT strain. More importantly, intracellular survival of the *∆usp76* mutant was assessed in CF patient‐derived macrophage cells and showed significantly reduced survival in four out of five CF macrophage preparations (Figure 7b) (0.05 > *p* > 0.0001).

## 4 DISCUSSION

A hallmark of the genus *Burkholderia* is its remarkable ability to survive and thrive in a range of diverse environments, ranging from the soil, aquatic niches, and disinfectants to the human host. Its ability to adapt to changing environments contributes to its success as a human pathogen. USPs are ubiquitous proteins that play a wide array of protective roles in bacterial pathogens and appear to be central to the pathogenesis of many intracellular pathogens, in particular (O'Connor & McClean, 2017). *B. cenocepacia* expresses 11 USPs. Six of these are encoded within the *lxa* locus, all of which showed increased abundance in chronic infection and increased expression in response to low oxygen (Cullen et al., 2018; Sass et al., 2013). Despite their upregulation in response to low oxygen and chronic infection, we now show that USP76, but not USP92, is involved in host cell attachment, highlighting a lack of redundancy in *B. cenocepacia* USPs. Moreover, we have shown that USP76 is likely to be important for the survival of *B. cenocepacia* in CF macrophages, a significant characteristic of this pathogen, and consequently may have a role in the chronic colonization of *B. cenocepacia* in people with CF.

To elucidate the role that USP76 plays in *B. cenocepacia* chronic infection, we examined a series of phenotypes associated with environmental pressures experienced during chronic infection or associated with Bcc virulence. The CF lung has profoundly low pO_2_ due to a combination of disease‐associated issues including mucous plugging, constant neutrophilic inflammation, and increased epithelial oxygen consumption (Mendelsohn et al., 2019; Montgomery et al., 2017). Oxidative stress in the CF lung contributes to the cycle of inflammation and is an inherent feature of CF (Scholte et al., 2019). Three key phenotypes relating to growth and/or survival under conditions typical of the CF lung were significantly altered in the ∆*usp76* mutant compared to the wildtype strain: hypoxia, low pH, and induced oxidative stress. It is clear that USP76 protects *B. cenocepacia* against these conditions and consequently facilitates survival in the CF lung. It should be noted that microbial communities in the CF lung are not exposed to uniform environments of pH and low oxygen, pH 4.5% and 6% O_2_ were chosen as representative stresses within the CF lung. UspA gene deletion mutants in *L. monocytogenes* also showed impaired growth when exposed to oxidative stress (Seifart Gomes et al., 2011). Similarly, *E. coli* UspA and UspD mutants were more susceptible to oxidative and superoxide stress (Nachin et al., 2005; Nyström & Neidhardt, 1994). *Listeria monocytogenes* UspA mutants exposed to acidic stress were also previously found to have reduced cellular growth (Seifart Gomes et al., 2011), while an *A. baumannii* UspA mutant was also more susceptible to both low pH and oxidative stress (Elhosseiny et al., 2015). Further, overexpression of a mycobacterial USP encoded by RV2624c increased survival in hypoxic conditions (Jia et al., 2016). Preliminary data indicate that USP92 appeared to play a role in virulence in the acute infection *Galleria mellonella* model, with the *∆usp92* mutant strain showing a 3‐increase in LD50 (unpublished data). Moreover, USP92 may be required for growth in the presence of high salt, but not in the presence of oxidative stress (unpublished data).

Previous reports showing that the *L. monocytogenes* UspA protected against low pH, oxidative stress, and enhanced survival within murine macrophages (Seifart Gomes et al., 2011), led us to evaluate whether USP76 also contributed to the survival of *B. cenocepacia* in macrophages, particularly given that USP76 was also protective in oxidative stress and involved in host cell attachment. The impaired survival of the ∆*usp76* mutant in U937 macrophage cells and, crucially, our finding that survival was significantly impaired in 80% of the CF patient‐derived macrophage samples confirms that USP76 confers a clear survival advantage to *B. cenocepacia* inside macrophage cells. People with CF can have impaired macrophage function, which can lead to altered phagocytosis and the killing of bacteria (Leveque et al., 2017), consequently, the role of USP76 in uptake and survival in CF patient‐derived macrophages is particularly noteworthy. The finding that impaired survival of the ∆*usp76* mutant was not evident in all CF patient‐derived macrophages samples, is not unexpected given the heterogeneity of human macrophages (Turton et al., 2021) and it is likely that other host factors, including infection status, also play a role (Leveque et al., 2017). For example, the levels of IL‐8 secretion by CF macrophages depend on the patient's lung function and the severity of the patient's lung disease (Leveque et al., 2017; Simonin‐Le Jeune et al., 2013). It should be noted that while PBMCs were collected from both males and females with CF, we were unable to successfully culture sufficient macrophages from any female samples. The BCAM0276 gene was previously shown to be upregulated when *B. cenocepacia* strain K56‐2 was internalized in murine macrophages (Tolman & Valvano, 2012). This study now confirms that USP76 contributes to the survival of *B. cenocepacia* within the CF lung and within the CF macrophage. Moreover, our previously observed increase in abundance of USP76 in chronically colonized patients indicates that USP76 is very likely to be a major player in facilitating the long‐term survival of this pathogen within macrophages of CF patients.

Different roles for individual USPs expressed by a bacterial species have previously been observed in *E. coli*. Opposing roles in attachment have previously been reported for individual USPs expressed in *E. coli*; with UspC and UspE mutants showing enhanced host cell attachment and loss of motility while UspF and UspG mutants had reduced host cell attachment and maintained cell motility (Nachin et al., 2005). Moreover, *E. coli* UspA and UspD were required for protection against oxidative stress while UspC and UspE were not. As with USP76, *E. coli* USPs have also been linked to cell adhesion when mutants were assessed by yeast agglutination, with mutants of UspC and UspE enhancing cellular attachment, UspF and UspG mutants reducing attachment (Nachin et al., 2005). The identification of surface arginine residues on USP76 and not USP92 suggested that these were likely to play a key role in host extracellular matrix attachment, via electrostatic interactions with sulfonate and carboxylate groups heparan sulfate on the surface of epithelial cells. It is well established that interactions of heparan sulfate with proteins are primarily driven by ion pair interactions mediated by surface regions rich in lysines and/or arginines, as in the case of the Heparin‐binding hemagglutinin (HBHA) protein expressed by *M. tuberculosis* (Esposito et al., 2012; Huang et al., 2017; Joseph et al., 2018). Heparan sulfate is reported to be the most important glycosaminoglycan in bacterial binding in CF cells, including Bcc (Martin et al., 2019). The lack of a reduction in binding of the ∆*usp76* mutant following heparanase treatment, in contrast to all other strains examined (all of which express USP76), supports the suggestion that the surface arginines on USP76 interact with the glycosaminoglycan and facilitate host cell attachment. Interestingly, macrophage cells increase expression of heparan sulfate under chronic inflammatory conditions, which would confer additional advantages on Bcc (Swart & Troeberg, 2018). Our working model is that USP76 enhances attachment of *B. cenocepacia* to host cells and confers a major advantage in its survival in the CF lung and increasing colonization fitness. The exact interaction between USP76 and the human cell surface is the subject of ongoing studies.

A question remains as to how a protein such as USP76, which is predicted to be cytosolic, mediates host cell attachment at the cell surface and can also mediate survival in stressful conditions such as low oxygen, acidic environments, or within macrophages. This multiplicity of roles has been observed with *E. coli* USPs previously, with UspF and UspG playing roles in both oxidative stress and host cell attachment (Nachin et al., 2005). In the case of these *E. coli* USPs, their host cell attachment role is associated with fimbriation. We cannot exclude a similar indirect effect in Usp76‐mediated attachment to host cells. However, it should be noted that there is increasing evidence of bacterial proteins with moonlighting functions, that show intracellular enzymatic functions and additional functions as receptors or adhesins to host proteins or tissues (Henderson & Martin, 2011; Wang et al., 2014). A key focus is establishing how USP76, which is predicted to be a cytosolic protein, is present on the bacterial cell surface. It is possible that consistent with many other predicted cytosolic proteins including many *E. coli* proteins (Monteiro et al., 2021), USP76 has an extracytoplasmic location. Recent work on O157 *E. coli* strains showed that 16, 14, and 31 predicted cytosolic proteins were located in the proteosurfaceome, proteovesiculome, and exoproteome respectively (Monteiro et al., 2021). OMV release by *Vibrio cholerae* in response to stress plays a comparable role in surface adaptation in vivo and colonization (Zingl et al., 2020), but this hypothesis and the potential release of USPs in outer membrane vesicles (OMVs) in response to stress will need to be further evaluated in *B. cenocepacia*. There have been over 300 moonlighting proteins identified to date (Chen et al., 2018), and research studies to elucidate this multifunctionality at multiple locations are yet to come.

In summary, USPs are critical for an environmental bacterium such as *Burkholderia* to cope with the breadth of stressful conditions that it is exposed to in soil. It is now clear that USPs also confer substantial advantages on opportunistic pathogens adapting to the niche environment of the CF lung and in particular, that USP76 is a major contributor to intramacrophage survival. Furthermore, the enhanced expression of USP76 throughout persistent infection indicates that it mediates, at least in part, the adaptation to chronic infection and represents an interesting target to overcome chronic colonization.

## AUTHOR CONTRIBUTIONS

**Andrew O'Connor**: Formal analysis (lead), Methodology (lead), Writing–original draft (lead), Writing–review and editing (equal). **Irene Jurado‐Martín**: Methodology‐Supporting, Writing–review and editing (supporting). **Margaritha M. Mysior**: Methodology (supporting), Writing–review and editing (supporting). **Anotidaishe Manzira**: Investigation (supporting), Visualization (supporting). **Joanna Drabinska**: Investigation (supporting), Methodology (supporting), Visualization (supporting), Writing–review and editing (supporting). **Jeremy C. Simpson**: Methodology (supporting), Resources (supporting), Supervision (supporting), Writing–review and editing (supporting). **Mary Lucey**: Resources (supporting), Writing–review and editing (supporting). **Rita Berisio**: Formal Analysis (supporting), Methodology (equal), Visualization (equal). **Kirsten Schaffer**: Resources (supporting), Writing–review and editing (supporting). **Siobhán McClean**: Conceptualization (lead), Funding acquisition (lead), Supervision (lead), Writing–original draft (equal), Writing–review and editing (lead).

## CONFLICT OF INTEREST

None declared.

## ETHICS STATEMENT

Full ethical approval was granted to KS for use of CF patient PBMCs on April 4, 2018 from the St Vincent's University Hospital Research Ethics committee. UCD Research ethics committee's full ethical approval was granted on May 9, 2018 for the sampling of blood from healthy volunteers (LS‐18‐47‐OConnor‐McClean).

## Figures and Tables

**Figure A1:**
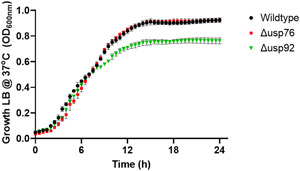
Growth of wild‐type, Δ*usp76* and Δ*usp92 mutant* strains over 24 h incubation in LB at 37°C with shaking. Data shown represent the mean OD_600nm_ ± standard deviation.

## Data Availability

All data are provided in full in the results section of this paper.

## APPENDIX 1

**Table A1:** Bacterial strains and plasmids used in this study

	Characteristics	Source
**Strain**		
***Burkholderia cenocepacia***		
K56‐2	ET12 Clone related to J2315	CF Clinical isolate, Canada (epidemic strain)
K56‐2 Δ*usp76*	Deletion of BCAM0276 in K56‐2	This study
*K56‐2* Δ*usp76_usp76*	BCAM0276 integration in K56‐2 Δ0276, Gn^S^	This study
K56‐2 Δ*usp92*	Deletion of BCAM0292 in K56‐2	This study
**Plasmids**		
pGPI‐SceI‐2	*ori*_R6K_, ΩTp^r^, mob+, containing the ISce‐I restriction site	(Flannagan et al., 2008)
pRK2013	*ori*_COIEI_, RK2 derivative, Kan^r^, mob+, tra^+^	(Figurski & Helinski, 1979)
pDAI‐SceI	Encodes the ISce‐I homing endonuclease, *ori*_pBBR1_, Tet^R^, P_dhfr_, *mob*+	(Flannagan et al., 2008)
pMH447	pGPI‐SceI derivative used for chromosomal complementation, Tp^R^	This study (as per Hamad et al., 2012)

**Table A2:** The primers used in this study, underlined sequences indicate restriction sites

Primer no.	Primer description	5**′** to 3**′** Primer sequence	Restriction enzyme
2761	BCAM0276 Upstream	TTGAGAATTCAAAGTCACTCGGCTACCC	*ApaI*
2762		AATTATCGATGAACAGCCGTGCGCC	*HindIII*
2763	BCAM0276 Downstream	TTTAATCGATGCTCCTCGGCAGTGTCG	*HindIII*
2764		AAAAGCTAGCGCGGCCGATCACGTACAGAA	*NheI*
2765	BCAM0276 Internal Gene	CAGTAGACGGTAGTCCTTCATCAAAAA	
2766		GATATCCGTCTCGACAATTTCCGTCTC	
2767	BCAM0276 Complementation	TTTTCATATGGAAACACATTCGAACGCC	*NdeI*
2768		GAAATCTAGATCCGGCCCGCACGTTC	*XbaI*
2921	BCAM0292 Upstream	TCCGGAATTCTGTCGGGTGACGCGCT	*EcoRI*
2922		GCTATAAGCTTCCGTCGAGCGCAACC	*HindIII*
2923	BCAM0292 Downstream	CAAGAAGCTTCGAGGCCACGGCTGC	*HindIII*
2924		GGCAGCTAGCCGTCCTCGACGAAAT	*NheI*
2925	BCAM0292 Internal Gene	AAGGATTACGCGTGACCGAG	
2926		ACGGTTCCTTTTCGGTGGAT	
4019	pGPI‐SceI/sequencing	GAGTGACACAGGAACACT	
4021		GCTCAATCAATCACCGGATCCC	
5386	pMH447 Construction	GGATCGAATTCCGGCTCGCCGCATTCCTGCAGG	*EcoRI*
5387		TCCGATCCATATGGTTCTGACCTCGGATCATTCG	*NdeI*
5388		CTCGCCAGCTAGCGCCGATATAGTCGGAGCCGAACATC	*NheI*
5389		TAGTGCTCATATGGTCGTTTCTAGATGCTCACGCAACTGCCGACCGC	*NdeI/XbaI*
5885	pMH447 Sequencing	TTGATGGCGAGCGATTCTTC	
5886		CCAGTTCTTCAGCGTGACGA	
3001	Confirmation of PC3 plasmid	AACAGCAACAACACCAAGTA	
3002		TTGCTTGCCTTCTTCTTCG	
3003		ACCCATTTATGGGAGCACAG	
3004		TGCTCCAGCATCACTTTCAC	
3005		CGGGCTGAACAGTAACAATA	
3006		GCTTGCTCGCTTTCTTTTTC	

**Table A3:** EPS production score of the WT and the Δ*usp76* mutant assessed on YEM agar plates

Strain	EPS Score (−/+/++/+++/+++d)	Description
WT	+	Some evidence of EPS
Δ*usp76*	+	Some evidence of EPS
WT at 6% O_2_	+	Some evidence of EPS
Δ*usp76* at 6% O_2_	+	Some evidence of EPS

*Note*: EPS production score of the WT and the Δusp76 mutant strains assessed following incubation on YEM agar plates for 5 days at 37°C on three independent occasions. EPS scored according to the method described by Zlosnik et al. (2008).

Abbreviations: EPS, exopolysaccharides; WT, wild‐type; YEM, Yeast extract mannitol.

## APPENDIX 2

see: Figure A1

## APPENDIX 3

Additional methods

**Assessment of antibiotic susceptibility**

Minimum inhibitory concentrations (MIC) were determined using Ceftazidime (0.016–256 μg), Levofloxacin (0.002–32 μg), Meropenem (0.002–32 μg), Polymyxin B (0.064–1024μg) ETEST® strips (Biomerieux). Overnight cultures were diluted to OD_600nm_ of 0.1 in sterile PBS and 100 μl aliquots spotted onto individual Mueller Hinton (MH) agar plates and using a PBS‐wetted sterile cotton swab, the cultures were then spread horizontally, vertically, and along the edge of the MH agar plates. Each ETEST® was placed in the center of the individual MH agar plates and incubated at 37°C for up to 48 h. The MIC value for each strain was determined by examining the elliptic inhibition zone intersecting the MIC value scale. The susceptibility of the strains to Polymyxin B was further examined by diluting overnight cultures 1:100 in LB and adding 270 μl to each well in duplicate in a 96‐well plate. A series of Polymyxin B concentrations, prepared in distilled water with 0.2% (w/v) BSA and 0.02% (v/v) acetic acid were serially diluted 1:10 to give a final concentration in the range of 16 to 1024 μg/ml and the plate incubated in a Biotek Synergy H1 Microplate reader at 37°C with medium shaking and OD_600nm_ measured every 15 min for 20 h (Loutet et al., 2015).

**Assessment of mucoid phenotype**

Exopolysaccharide (EPS) production was assessed to determine the mucoid phenotype of the mutant strains of Bcc using Yeast extract mannitol (YEM) (2 g/L Yeast extract, 20 g/L d‐mannitol, 15 g/L Agar no.2) plates (Lagatolla et al., 2002). Overnight cultures of each strain were streaked onto YEM plates in a quadrant streak manner and incubated at 37°C for 5 days in normal oxygen conditions or low oxygen conditions using a controlled hypoxic chamber (COY). EPS production and mucoid phenotype were scored as follows: The scoring of mucoid phenotype was as follows: (−) bacterial colonies on YEM plates appeared dry with a matte appearance and showed no evidence of EPS production, (+) bacterial colonies predominately non‐mucoid with some evidence of EPS production in the confluent area of growth on the YEM plate, (++) mucoid phenotypes with EPS production on both the confluent area of growth and streaked individual colonies which appeared flat on the YEM plates, (+++) excessive EPS production with a poor distinction between confluent area and streaked area, streaks appeared raised due to EPS production; a score of (+++d) was similar to (+++) but the EPS production resulted in drips of EPS on the lid of the Petri dish when inverted (Zlosnik et al., 2008).

**Determination of motility**

Motility assays were performed on each mutant strain to determine the effect of gene deletion on the ability of swimming, swarming, and twitching (Cullen et al., 2015). All agar plates were poured on a flat, even surface and allowed to dry for 30 min aseptically under laminar flow. Swimming motility was assessed by inoculating the surface of the agar plate (0.3% w/v LB agar) with 5 μl of an overnight culture using a sterile yellow 20 μl pipette tip. Plates were wrapped in parafilm, inverted, and incubated at 37°C for 48 h after which the average of two diameter measurements of the zone motility at 24 and 48 h was measured. Strains were scored as follows: nonmotile (diameter ≤5 mm), motile (diameter >5 and ≤60 mm) or highly motile (diameter of >60 mm). Swarming motility was assessed by piercing 5 μl of an overnight culture into the center of the (0.5% w/v LB agar) with a sterile yellow 20 μl pipette tip without piercing through the agar. Plates were wrapped in parafilm, not inverted, and incubated at 30°C for 48 h and the average of two diameter measurements of the zone motility at 24 and 48 h was determined. Strains were scored as follows: nonmotile (diameter ≤5 mm), motile (diameter >5 and ≤60 mm), or highly motile (diameter of >60 mm). Twitching motility was assessed by inoculating the agar (1% w/v LB agar) with 5 μl of an overnight culture as above and incubating at 37°C for 24 h. Plates were removed from the incubator and dried under laminar flow for 30 min, the agar was removed before staining the plates with 0.5% w/v Coomassie brilliant blue R250, 40% v/v Methanol, and 10% v/v acetic acid for 2 min. The excess stain was then washed off with a solution of 40% methanol and 10% acetic acid. Twitching was determined by taking the average of two diameter measurements of the zone motility. Strains were scored as follows: nonmotile (diameter ≤5 mm), motile (diameter >5 mm and ≤30 mm) or highly motile (diameter of >30 mm).

**Assessment of biofilm formation**

Overnight cultures (10 ml) of each strain were transferred to 100 ml of fresh LB and incubated at 37°C for 4–5 h with agitation at 200 rpm. OD_600nm_ was measured and cells were diluted to ~ 1.6 × 10^6^ CFU/ml in LB. Each strain (200 μl) was added to each of the eight wells in the respective columns of a triplicate 96‐well round bottom plate with sterile LB used as the negative control. Plates were incubated at 37°C in normoxic or hypoxic (6% O_2_) conditions for up to 72 h. Plates were removed at 24, 48, and 72 h intervals, and wells were washed twice with sterile water before the addition of 125 μl of 0.1% w/v crystal violet solution to stain biofilm mass for 30 min. The wells were washed three times with sterile water and allowed to dry for 30 min at RT, before the addition of 100 μl of 95% v/v ethanol to solubilize the stained biofilm for 15 min. The solution was transferred to a fresh 96‐well flat bottom plate and the OD_590nm_ was measured. Wells with an OD reading of ≤0.120 are considered non biofilm forming. OD readings of ≥0.120 but <0.240 are considered poor biofilm formation and OD readings of >0.240 are strong biofilm formation.
